# Synthesis by solid route and physicochemical characterizations of blends of calcium orthophosphate powders and mesoporous silicon particles

**DOI:** 10.3389/fbioe.2023.1101513

**Published:** 2023-03-20

**Authors:** Caroline Richard, Mélissa Alfred-Arulrasa, Harikrishnan Ramadas, Pubudu Tharanga Mahagamage, Thomas Defforge, Gael Gaultier, Cécile Autret-Lambert, Nathalie Poirot, Eric Champion, Amandine Magnaudeix

**Affiliations:** ^1^ GREMAN UMR CNRS 7347, Insa Centre Val de Loire, Université de Tours, Tours, France; ^2^ Indian Institute of Technology of Kharagpur, Kharagpur, India; ^3^ Institut de Recherche sur Les Céramiques (irCer), UMR CNRS 7315, Université de Limoges, Limoges, France

**Keywords:** hydroxyapatite (HAP), tricalcium phosphate (TCP), biocompatibility, ball milling method, mesoporous silicon

## Abstract

The purpose of the study was to investigate the synthesis of economic calcium phosphate powders from recycled oyster shells, using a ball milling method. The oyster shell powder and a calcium pyrophosphate powder were used as starting materials and ball milled, then heat treated at 1,050°C for 5 h to produce calcium phosphate powders through a solid-state reaction. Electrochemically synthesized mesoporous silicon microparticles were then added to the prepared phosphate powders by mechanical mixer. The final powders were characterized using X-ray diffraction, Fourier transform infrared spectroscopy, and scanning electron microscopy to analyze their chemical composition and determine the most suitable process conditions. The biocompatibility of the produced powders was also tested *in vitro* using murine cells and the results showed good biocompatibility.

## 1 Introduction

Every year millions patients worldwide sustain bone grafting procedures to repair bone defects stemming from diseases (cancer, infections, osteoporosis …) or traumatic events. Mammalian bone tissue is composed of about 70 wt% inorganic mineralized calcium phosphate in the form of biological hydroxyapatite (HAp). A first experiment of the use of calcium phosphate for a fracture treatment dates back a century (Habraken et al., 2016; Bobbio, 1972). Bone-like chemical composition, microporosity, specific surface area, crystallinity, crystal size, and surface roughness are important factors to consider, as are the properties of autologous and allogenic bone grafts. Thus, the ideal bone substitute (Jordana et al., 2017) should be biocompatible, bioresorbable, easy to use, inexpensive, while exhibiting i) a structural integrity similar to bone, ii) an osteoconductive matrix, and iii) contain osteoinductive factors, capable of recruiting and stimulating osteoforming cells for bone repair and regeneration. Bone tissue engineering is based on the synergy between an appropriate substitution biomaterial and cells (Jordana et al., 2017). Hydroxyapatite (HAp–Ca_10_(PO_4_)_6_(OH)_2_), Tricalcium Phosphate (TCP–Ca_3_(PO_4_)_2_) and Biphasic Calcium Phosphates (BCP, HAp + TCP mixtures) are the commonly used biomaterials in bone surgery. Most of the time, these compounds are of synthetic origin. However, biological or organic HAp can be found from heat-treated bovine cancellous bone. Recent studies have managed to create lower size nano hydroxyapatite by sol-gel method. Nano hydroxyapatite generated more interest in bone tissue engineering because of its size, similar to bone cells (Habraken et al., 2016). Mainly used for filling, HAp is osteoconductive by providing a support for bone cells and bone morphogenetic proteins. In contact with healthy bone, osteoid tissue is formed and then mineralized, with the progressive acquisition of a mechanical strength similar to that of bone. HAp can be also employed in various applications in medical industry: HA coated titanium and stainless-steel parts often used in clinical applications. These HA coated metal parts show low rejection rate and higher biocompatibility. Tooth Enamel contains 97%wt of nano-hydroxyapatite. A recent study shows that nano-hydroxyapatite was successfully used in enamel repair (Habraken et al., 2016).

TCP (Ca/P = 1.5) has two crystalline forms: ɑ-TCP and β-TCP. TCP is considered as the first resorbable bioceramic. β- TCP is the most used and can be converted to biological HAp *in vivo*. Wu et al., 2011a reported the order of the relative solubility of the bioceramics as ɑ-TCP > β-TCP >> HAp; Dorozhkin, (2009) pointed out TCP cannot be precipitated from aqueous solutions. The formation of β-TCP takes place at about 1,050°C in the solid route process for example from an equimolar blend of CaCO_3_ and Ca_2_P_2_O_7_. TCP can be also formed by thermal decomposition above 700°C of a precursor Ca-deficient hydroxyapatite obtained by aqueous precipitation with proper Ca/P of 1.5, also called apatitic tricalcium phosphate (Destainville et al., 2003).

The BCPs combine the physicochemical properties of each component. This allows a controlled bioactivity by modulating the HAp/TCP ratio.

Since the beginning of the 2000s, numerous studies demonstrated the capacity of the nacre from molluscan shells as an interesting natural substitution biomaterials *versus* the usually used synthetic biomaterials (Chen et al., 2016; Brundavanam et al., 2017; Jordana et al., 2017).

The Pacific oyster or Japanese oyster, *Magallana Gigas* or. *Crassostrea Gigas* (Thurnberg, 1793) was introduced in France at the end of the 1960s. This oyster is distinguished from those of most bivalves by a very flaky appearance, and by its material. It is made of almost pure calcite: 91–95%wt CaCO_3_ (containing about 40% of Calcium), 4%wt organic matter and small amount of oxides (Ho et al., 2021; Rujitanapanich et al. (2014)). Ramakrishna et al., 2018 stated that the oyster shell is usually composed of 90% calcite and 10% aragonite (aragonite transforms to calcite very slowly at room temperature). This oyster is widely farmed, especially in France, to such an extent that it represents 75% of the European production but only 2% of the world farming production (more than 4 million tons per year). This makes it the most commercially important tasting oyster species, with a world market share of 93.7% (Silva et al., 2019). Each year, oyster breeding in France generates about 140,000 tons of waste products. The major French oyster farming regions (Normandy, Brittany, Occitania.) have quite recently started to organise the collection of this biogenic waste with high diversified recycling potential.

The use of bone substitutes is constantly increasing thanks to the recent development of injectable forms. Several bone substitutes for surgical indications, combining growth factors and osteogenic cells to induce true bone tissue regeneration have been investigated or are proposed. It is often a question of adding a certain number of organic or inorganic compounds to boost osteoinduction (as PRP—Platelet Rich Plasma and PFR—platelet Rich Fibrin), or to limit the risks of sepsis (with Silver or Strontium) (Faiz Mohd Yusoff et al., 2021). Innovative bioceramics can be developed with association of Silicon for example. Silicon is a trace element found in the body. Although it is not an essential trace element, it is important for the immune system and for maintaining good bone health. Indeed, Silicon plays a role in the binding of calcium or bone calcification and is found in the osteoid border where bone is built (Camaioni et al., 2015). The release of silicon ions contributes to increase the multiplication and the differentiation of bone cells for a healing of shorter duration (Salonen and Lehto, 2008). Chadwick et al. (2010) indicated the first studies in 1995 concerning the dissolution of porous silicon (PS) in SBF (Simulated Body Fluid). They showed and confirmed the growth of HAp on the nanoporous silicon particles and proved that PS can be a real bioactive material. In the same way, Bowditch et al. (1998); Low and Voelcker. (2014); Dalilottojari, et al. (2015) reported the biocompatible, biodegradable and bioactive properties of PS with size pore tunability, high surface area, and opened outlooks in different fields as tissue engineering, drug delivery, oncology, orthopedics, ophthalmology and even *in vivo/in vitro* biosensors or biomarkers (Hernández-Montelongo et al., 2015).

As HAp is the most widely used biomaterial for bone repairs, various techniques have been developed to produce HAp powders. Two main routes are possible: wet route and solid-route. Different processes are possible as: solid-state, mechanochemical, conventional chemical precipitation, hydrolysis, sol-gel, hydrothermal method, emulsion method, sonochemical method, combustion and even pyrolysis. Zaman et al., 2020 have identified the advantages and disadvantages of each method. Nevertheless, the solid-state method is less frequently reported although it is relatively simple and inexpensive compared to the wet treatment methods. The major advantage of solid state reaction method is that the reactions are faster and there is no need to adjust the pH value as in the case of precipitation and other wet methods (Ho et al., 2013). It could be an effective route to obtained nanosized HAp and control of the size and morphology of the particles. The disadvantages mainly concern the control of the purity (contaminations) and, the formation of agglomerates. Rhee (2002) and Wu et al., 2011b demonstrated the feasibility of producing HAp from oyster shell powder by a detailed mechanochemical technique but without biocompatibility tests.

Our investigation focused on various synthesis conditions of HAp from recycled oyster shells (calcination temperature, heat treatment duration, milling time) using a mechanochemical way and solid state reaction to produce BCP powders from oyster shells. In order to improve the biological behaviour and extend the potential applications, addition of mesoporous silicon particles as source of silicon and possible drug inclusion in their pores, was also investigated. *In vitro* assays using a MC3T3-E1 pre-osteoblast cell line were carried out to investigate the biological behaviour of the materials.

## 2 Materials and methods

### 2.1 Oyster shell powders synthesis *via* solid route

#### 2.1.1 Raw powder

Oyster shell powders (OSP) were supplied from Ovive SA (France), an industrial actor committed to the environment both through its actions of recycling shellfish waste. The origin of oyster shells is the Atlantic coast of France. The powders were specially conditioned (cleaned, dried and then crushed by Ovive SA) to produce the final product for medical applications. The particle size was in the range of 300–400 μm. This narrow size distribution was selected for more uniformity.

#### 2.1.2 Chemical-physical characterization techniques

SEM and XRD analysis were conducted for OSP and for the produced calcium phosphate powders. X-ray diffraction (XRD) analysis was carried out using a Bruker D8 Advance Diffractometer to examine the phase purity, crystallinity and lattice parameters of calcium phosphate powders. XRD patterns were collected from 2θ: 20°–60° with steps of 0.02° using a Cu- Kɑ radiation (λ= 0.154 nm). Powder diffraction files (PDF, detailed in Figures 2, 5) from the International Centre for Diffraction Data (ICDD) were employed as the reference patterns to identify the crystalline phases. Microstructure observations of the initial powders and treated samples were done using Scanning Electron Microscope (SEM) (ZEISS Gemini series, Germany). The powders were coated with a 5–8 nm layer of gold-palladium using a Precision Etching System (Gatan Ametek, United States) to improve imaging. The elemental chemical compositions of OSP and also of the sintered powders were determined by Energy Dispersive X-ray spectroscopy (EDS) analysis.

FT-IR analysis was conducted on raw oyster shell powder samples using a Jasco model 4,600 (Japan) apparatus with the HATR (Horizontal Attenuated Total Reflectance) technique and a compression cell. The samples were analyzed in the range of 500–4,000 cm^−1^. The powders were mixed with KBr and pressed into pellets for analysis. A thermal analysis TGA/DTA was performed using a STA regulus 2,500 (Netzsch, Germany) facility under a nitrogen atmosphere with a heating rate of 10°C/min from 20°C to 1,000°C.

Granulometry analysis was performed with a Zetasizer Nano-ZS (Malvern Panalytical, Netherlands, red Laser) by Dynamic Light Scattering (DLS) in alcoholic medium (isopropanol) at room temperature. Hydrodynamic diameters of the particles were determined by Stokes-Einstein equation. Refractive index was 1.44 for porous silicon (Astrova and Tolmachev, 2000) and 1.67 for HAp.

#### 2.1.3 Preparation of calcium phosphates

The as received OSP was mixed with calcium pyrophosphate (Ca_2_P_2_O_7_) supplied by Santacruz Biotechnology (France) in the molar ratio 4:3 (Ca/P ratio in stoichiometric HAp). For the synthesis of approximatively 10 g of sample, 7.59 g of Ca_2_P_2_O_7_ and 3.98 g of OSP were mixed with 8 mL of deionized water to avoid wear and debris of stainless steel balls and surface of stainless steel container during milling process. A Fritsch pulverisette 6 (classic line) planetary ball milling machine was used for milling the samples. 100 g of Fritsch stainless steel balls (5 mm diameter) were employed so that the weight ratio of powder to that of balls remains at 1:10 (capacity of container: 80 mL). The samples were milled for 18 h at 3 RCF (g). The obtained pastes were then dried in an oven at 150°C for 3 h. The dried pastes were heat treated, at a rate of 10°C/min, at 1,100°C during 5 h. Then they were milled at 4.5 RCF (g) during 1 h to pulverize them. The obtained powder was coded as M18H5. In addition, to facilitate the mechanical milling process and eliminate aggregates, the dried pastes were ground by hand in an agate mortar both before and after the milling step.

The choice of the temperature and duration of heat treatment were determined by literature review and previous studies. Increasing the temperature of the heat treatment increases generally the amount of HAp. The ideal temperature for the synthesis is set between 1,000°C and 1100°C (the formation of β-TCP takes place at about 1,050°C). For the experiments, the temperature was chosen to optimize the formation of HAp and β-TCP. A long heating time allows the complete transformation of CaCO_3_ and Ca_2_O_7_P_2_ for a final product without impurities. According to literature, the heating time can vary between 3 and 5 h depending on the source. Therefore, the longer time of 5 h was chosen.

#### 2.1.4 Preparation of mesoporous silicon (MePS) particles

Mesoporous silicon was produced by using the electrochemical etching technique. The cell was made of two platinum electrodes placed on each side of a p-type silicon wafer (10–20 mΩ·cm) that was oriented in the (100) direction and immersed in an aqueous solution containing hydrofluoric acid (HF). The polarization of the electrodes enabled the one of the wafer, defining the anodic (porous) and cathodic sides. The electrolyte consisted of 30% HF, 25% acetic acid, and 45% deionized water (in weight). The anodizing treatment was performed at a constant current density of 30 mA/cm^2^ for 90–120 min with the goal of creating mesoporous silicon layers with average pore sizes ranging from 5 to 50 nm (Uhlir, 1956; Herino et al., 1987; Lehmann et al., 2000). After the etching process, the wafer was rinsed with water and then isopropanol (IPA) to remove any residual electrolyte from the porous structure. It was then placed in an ultrasonic container for 12 min to separate the porous layer from the parent substrate. The resulting millimeter-scale particles were removed from the container, placed in a paper filter, and rinsed with IPA for 20 min. The particles were stored in IPA and then ball-milled for 30 min in a Fritsch Pulverisette 6 ball miller using 4.5 RCF (g) of stainless steel balls with a diameter of 10 mm. The micro- and nanoparticles were then separated by size using a Hettich Universal 320 centrifuge at two different rotation speeds (40 RCF (g) and 3985 RCF (g) for 10 min each) to remove the largest and smallest particles, respectively. The final step was the evaporation of the IPA.

#### 2.1.5 Preparation of the blend of calcium phosphates and MePS

M18H5 powder and MePS particles were mixed manually with a spatula for 1-2 min, in a ratio of 70% M18H5 to 30% MePS in accordance with the considerations for organic glasses (Lao and Nedelec. 2014). The mixture was preserved in alcoholic solution. Then, this mixture was dried at 150°C for 3 h.

### 2.2 Evaluation of the biological properties

#### 2.2.1 Cell model and *in vitro* cell culture

The MC3T3-E1 subclone 14 cell line (CRL2594^®^, ATCC, United States, then referred as MC3T3-E1) was used as cell model. These cells are murine pre-osteoblasts derived from newborn calvaria and were selected for their ability to differentiate homogeneously in mature osteoblasts able to produce a mineralized extracellular matrix. These cells were routinely cultured in a non-differentiating culture medium in an incubator heated at 37°C under 5% CO_2_ and wet atmosphere. The culture medium named after “complete culture medium” was composed of alpha modified Eagle’s medium, without ascorbic acid containing L-glutamine (ɑMEM, A1049001, Gibco, United States) and supplemented with 10% fetal calf serum (FBS, 10270106) and 100 μg/mL streptomycin, 100 U/mL penicillin (BP2959-50, Fisher Scientific, United Kingdom).

The medium was replaced every 2-3 days and cells were passaged when the cell confluence reached 70%–80% of the culture area. After having discarded the cell culture medium, the cell layer was rinsed in 1X phosphate buffer saline (PBS, 141900094, Gibco, United States). It was then covered by TrypLE select (12563029, Gibco, United States) for 3 min to allow cells to detach from culture plasticware. The cells were harvested in complete culture medium and centrifuged at 300 *g* for 5 min at room temperature. The cell pellet was resuspended in complete culture medium and the concentration of viable cells was established by numeration of an aliquot in a Mallassez’ hematocytometer for further seedings.

#### 2.2.2 Preparation of the powder extracts

Three powders extracts were prepared in complete culture medium as described hereafter, according to the ISO EN 100993-5: 2010 standard. For standardization of the exchange surface with the solution, an amount of powder was weighed using a microbalance (Sartorius, Germany) to get an Extraction Ratio (ER) of 6 cm^2^/mL (<0.5 mm thick) in a final amount of solution of 100 mL. Powder extracts obtained from M18H5 powder were prepared according to its specific surface area of 45 m^2^/g. Regarding the M18H5/MePS mixture, two powder extracts, respectively named M18H5/MePS_1 and M18H5/MePS_2 were prepared according to the specific surface of M18H5 powder (45 m^2^/g—powder extracts named M18H5/MePS_1) or MePS (300 m^2^/g—powder extracts named M18H5/MePS_2—Table 1). The powders were conditioned in glass tubes closed by an aluminum foil and heated at 200°C for 2 h for sterilization. Under laminar flow hood, powders were resuspended in 1 mL of complete culture medium and thoroughly mixed. 250 μL of this suspension were immediately mixed into 24.75 mL of complete culture medium in 50 mL sterile conic centrifuge tube (Corning, United States). The solutions were incubated for 24 h at 37°C under 300 rpm agitation.

**TABLE 1:** Powders used and their assumed surface specific area for the preparation of extract used for biological assays.

Powder	Sample name	Assumed specific surface area (m^2^/g) for extract preparation
M18H5	M18H5	45
M18H5/MePS mixture	M18H5/MePS_1	45
	M18H5/MePS_2	300

The extracts were then characterized by their silicon content released from powders determined by Inductive Coupled Plasma—Optical Emission Spectrometry (ICP-OES)—OptimaDV8300, PerkinElmer, United States). The extract solutions were used either pure (100%) or diluted in complete culture medium at 25%, 50%, and 70%.

#### 2.2.3 Cell seeding and exposition to powder extracts

Cells were seeded at a density of 20,000 cell/cm^2^ in 100 µL of complete culture medium in 96-well plates (Sarstedt, Germany) for MTT assay, 24-well containing 12 mm borosilicate coverslip (ThermoFisher Scientific, United States) for assays using microscopy. The cells were then incubated at 37°C for 24 h to let the cells to adhere. The culture medium was then discarded and replaced by powder extracts or their dilutions for 24 h and 48 h.

#### 2.2.4 Evaluation of cell metabolic activity: MTT assay

After 24 h and 48 h of culture, 10 µL of 12 mM MTT 3-[4,5-dimethylthiazol-2-yl] −2,5 diphenyl tetrazolium bromide (Sigma-Aldrich, United States) were added to the wells and the cells were incubated for 2 h at 37°C to let the cellular dehydrogenases to metabolize the soluble yellow MTT into deep-purple insoluble crystals of formazan. The culture medium containing MTT was discarded and 50 µL of dimethyl sulfoxide (Sigma-Aldrich, United States) were pipetted onto the cells to lyse them and dissolve the formazan crystals. After 15 min of incubation at room temperature in the dark, under agitation, the optical density or absorbance was measured at 595 nm using a microplate reader (Fluostar Optima, BMG Labtech, Germany).

#### 2.2.5 Cell proliferation

For cells cultured on borosilicate glass coverslips, after 20 h of incubation, EdU (5-ethynyl-2′-deoxyuridine, thymidine ThermoFisher Scientific, United States) was added to the culture media for a final concentration of 10 µM per well. After 4 h of incubation at 37°C, cells were rinsed 3 times in PBS 1X and fixed in paraformaldehyde (PFA, Sigma Aldrich, Germany) 4%w/v in PBS 1X for 10 min at room temperature. After 3 washes in PBS 1X, cells were immersed in Tris 100 mM, pH 7.6 for 10 min at room temperature and permeabilized in 0,5%_v/v_ Triton-X100 (ThermoFisher, United States) in PBS 1X for 15 min at RT. A click-chemistry reaction solution was prepared by adding one by one as follows 887,5 µL PBS 1X, 10 µL 200 mM CuSO_4_ (Sigma Aldrich, United States), 2,5 µL AlexaFluor 488 azide (ThermoFisher Scientific, United States) and 100 µL 200 mg/mL ascorbic acid (Acros organics, United States), and incubated on cells in the dark for 30 min at RT. A counterstaining of nuclear DNA was carried out with 20 µM Hoechst 33,342 (ThermoFisher Scientific, United States) in PBS 1X for 5 min at RT in the dark.

#### 2.2.6 Cell morphology

After 24 h, cells cultured on borosilicate glass coverslips were rinsed 3 times in PBS 1X and fixed in PFA 4% for 10 min at room temperature. They were rinsed 3 times in PBS 1X and permeabilized for 15 min at room temperature in 0.1%_v/v_ Triton-X100 (ThermoFisher, United States) in PBS 1X. Aspecific antigenic sites were saturated by immersing the cells for 1 h at room temperature in Bovine Serum Albumin (BSA, Sigma Aldrich, United States) 3%_w/v_ in PBS 1X. Actin cytoskeleton was stained by incubated the cells in presence of Phalloidin conjugated to Dylight 488 (ThermoFisher Scientific, United States) for 1 h at room temperature in the dark. An immunolabelling of ɑ-tubulin was performed by hybridizing the cells first, with a monoclonal mouse anti ɑ-tubulin antibody (SantaCruz Biotechnology, United States) for 1 h at room temperature and, secondly, with a goat-anti-mouse antibody conjugated to Dylight 550 (ThermoFisher Scientific) for 1 h at room temperature. Nuclei were stained with Hoechst 33,342 as described above.

#### 2.2.7 Fluorimetric dosage of calcium in culture supernatants

After 24 h of cell culture in the powder extracts, the culture supernatant was collected and the calcium was dosed using a commercial fluorimetric dosage assay (Abcam, United Kingdom) according to the manufacturer’s instructions in black 96-well plates (Corning, United States). The fluorescence was measured in a plate reader (Fluostar Optima, BMG Labtech, Germany) with an excitation at 544 nm and emission at 590 nm.

#### 2.2.8 Microscopy image analysis and statistical assays

For each experiment needing to make microscopy observation, at least 10 fields were imaged using an upright AxioImager M2 fluorescence microscope coupled to an AxioCam MRm camera (Zeiss, Germany). Images were then analyzed using the ImageJ software. Cell density was measured by counting the Hoechst 33,342 positive nuclei reflecting the cell number and reported as number of cell/cm^2^. The proliferation rate was calculated as the percentage of EdU positive nuclei on Hoechst 33,432 positive nuclei.

For each test, at least 3 independent experiments were performed with freshly prepared powder extracts. Statistical analyses were done using GraphPad Prism 9. Data were assayed for normality using the Shapiro-Wilk test. According to the data set and the results of the normality test, either a Kruskal–Wallis test followed by a Dunn’s *post hoc* test (non-parametric) or an ANOVA one-way followed by a Tukey *post hoc* test was performed. Differences were considered as significant for *p* ≤ 0.05.

## 3 Results and discussion

### 3.1 Characterization of raw oyster shell powders

**FIGURE 1:**
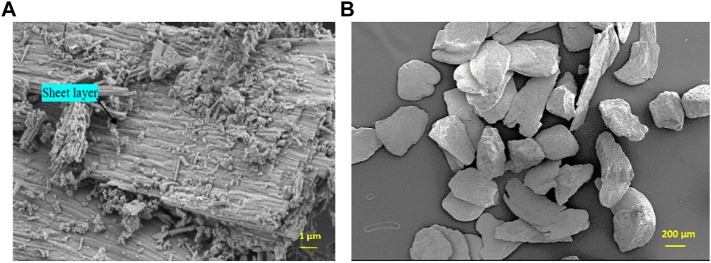
SEM images (SE mode) of selected raw oyster shell powder for the synthesis. **(A)** Detail of the surface of a particle (bar: 1 µm). **(B)** Global view and morphology of the particles (bar: 200 µm).

The SEM analysis provided the micrographies of the OSP particles. Figure 1A shows at high magnification details of the surface of a particle with typical outer prismatic layers or sheet layers of the shell. They are generally oriented to the growth direction of the oyster shell (Yoon et al., 2003). The surface is irregular with a lot of debris as short mineral fibers and Figure 1B. Shows the global morphology and size of the particles of the selected powder.

The Energy Dispersive X ray analysis (EDS) was also performed on the samples of OSP. The presence of Calcium (36.09%), Oxygen (47.25%), Carbon (14.43%) was evident. Elements like Magnesium (0.71%), Sodium, Silicon (1.53%) were also present in trace amounts. These elements could replace Calcium in the carbonate.

**FIGURE 2:**
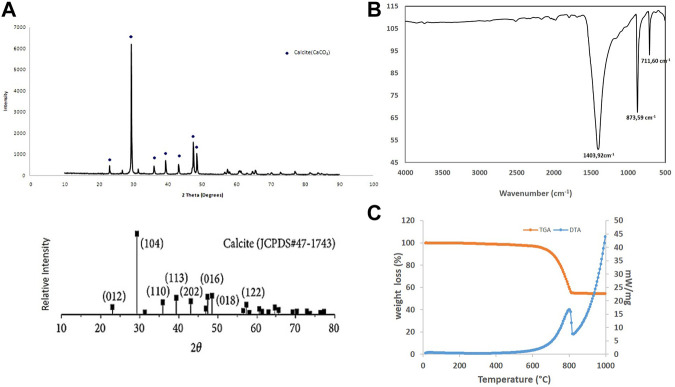
Physicochemical characterization of oyster shell powder. **(A)** X ray diffractogram compared with JCPDS standard with the diffracting crystal planes and **(B)** FTIR pattern with the characteristic carbonate vibrational bands. **(C)** TGA/DTA of raw oyster shell powder.

The Figure 2A. Shows the X ray pattern of raw OSP. Calcite is the major mineral component of the powder (JCPDS N° 47-1743)). No aragonite phase, other polymorphic biocarbonate, was detected. The presence of sharp diffraction peaks in the raw sample indicates high crystallinity (rhomboedric microstructure—R $\bar{3}$ m space group).

Characteristic FT-IR bands at 1,403.92 cm^−1^, 873.59 cm^−1^, 711.60 cm^−1^were noted (Figure 2B). 1,403.92 cm^−1^ and 873.59 cm^−1^ correspond to ν3-asymmetric CO_3_ stretching and ν1 asymmetric CO_3_ deformation respectively. 711.73 cm^−1^ is attributed to ν4 symmetric CO_3_ deformation. The 873.59 cm^−1^ and 711.60 cm^-1^ bands match closely with the calcite reference vibrational bands (Gunasekaran et al., 2006; Rujitanapanich et al., 2014) and confirm the composition of the raw oyster shell powder in calcite. The slight shifts may be attributed to impurities and/or distortion of carbonate groups.

Figure 2C presents the results of a TGA/DTA performed on raw OSP. The organic matter and humidity present in the sample were removed between 150°C and 200°C. An endothermic reaction, as shown by the DTA curve is recorded between around 700°C and 820°C. The calcination of CaCO_3_ was observed to decompose into CaO following the reaction CaCO_3_→CaO+CO_2_↑ (Choi et al., 2011; Hasan Mahmud et al., 2015; Cudrado and Rica, 2017). This measurement confirmed the chemical composition of the oyster shell powder.

### 3.2 SEM micrographies of the crushed OSP and M18H5 powder

**FIGURE 3:**
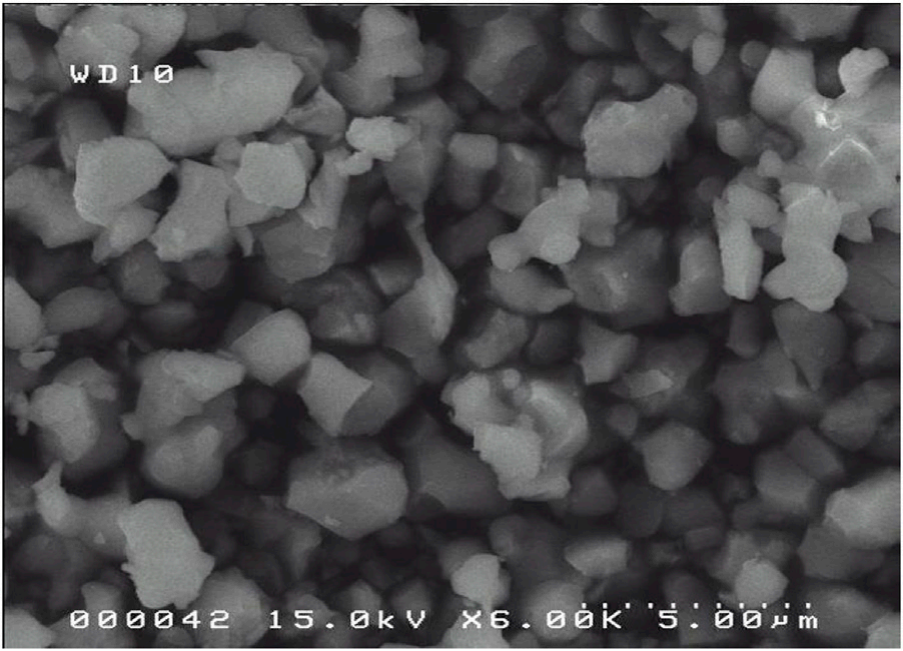
SEM images of crushed OSP sample (Milling duration: 18 h).

Figure 3 shows the crushed OSP (duration of milling: 18 h). The grain shape is rather uniform, the surface smooth, and few aggregates are present. The grain size is more or less homogeneous. By image analysis, the average diameter is 2.06 µm.

**FIGURE 4:**
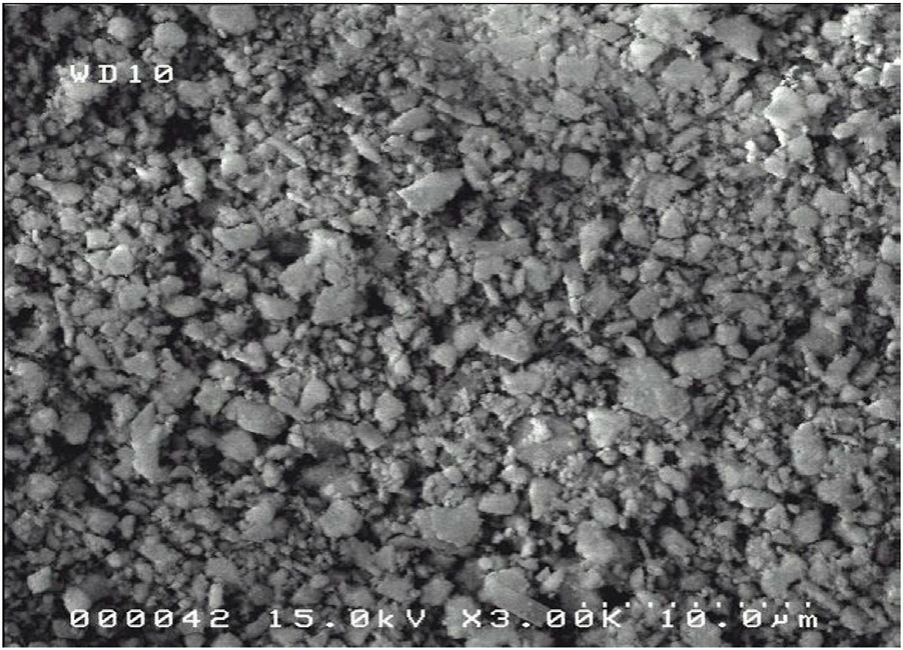
SEM Images of M18H5 sample.

After heat treatment, the grains agglomerate and their shapes are less regular than previously. The crystals are white and opaque. In addition, the surface of the grains looks rougher (Figure 4).

### 3.3 Calcium phosphates characterization

**FIGURE 5:**
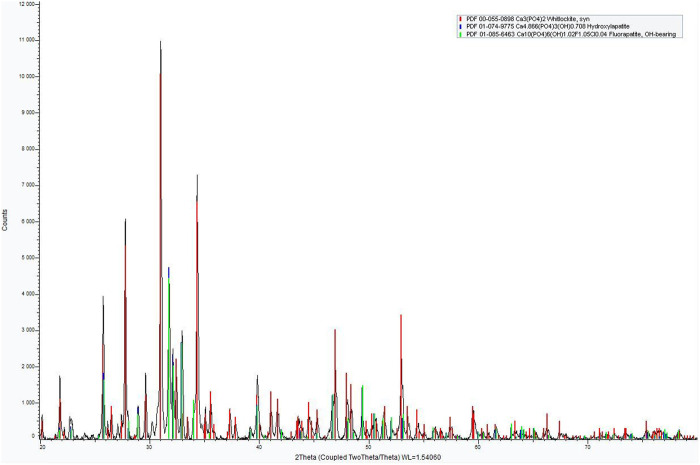
XRD pattern of M18H5 powder.

The X-ray diffraction analysis of the M18H5 powder is presented in Figure 5. The XRD pattern reveals the presence of a mixture of calcium phosphates, including β-tricalcium phosphate (TCP; PDF 00-055-0898) and calcium phosphate apatites that can be indexed as hydroxyapatite (HAp; PDF 01-074-9775), as illustrated in Figure 5.

**FIGURE 6:**
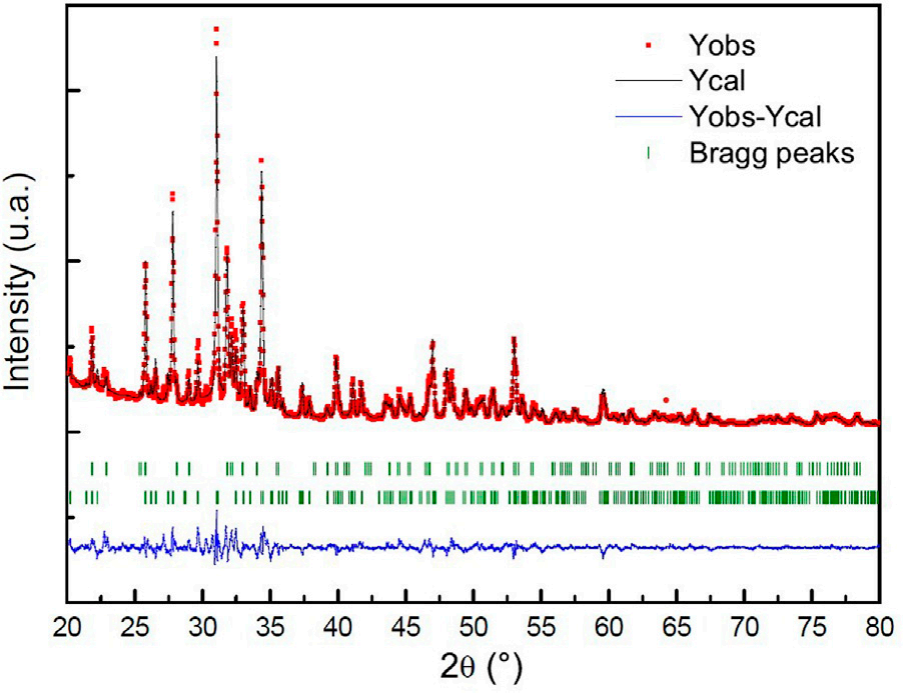
Rietveld analysis pattern of obtained powder. The black solid line concerns the calculated intensities and the red asterisk line, the observed intensities. The short vertical lines show the position of possible Bragg reflections. The difference between the observed and calculated intensities is plotted below the profile.

The powder is a Biphasic Calcium Phosphate (BCP). The XRD pattern indicated a majority of β-TCP. The quantification of the amount of each calcium phosphate is roughly estimated by a RIR (Reference Intensity Ratio) method with the two higher peak intensities of β-TCP and HAp as proposed by Raynaud et al., 2001 and in the works of Destainville et al., 2003. The results indicated 75 wt% for β-TCP, 25 wt% for HAp. To obtain a more precise determination, a Rietveld refinement was conducted using the Fullprof software, and the results are presented in Figure 6.

The Rietveld refinement method shows that 24.1% of the sample is composed of hydroxyapatite (HAp) and 75.9% is composed of β-tricalcium phosphate (β-TCP). The reliability factors for this structural refinement of the X-ray diagram, Rp and Rwp, are close to 7.32% and 9.42%, respectively. It should be noted that these values are somewhat high because minor phases present in the sample were not taken into account in the refinement due to their low proportion. Some lines in the X-ray diagram are not indexed, including broad lines around 24° and two peaks at 27.12° and 30.13°. It is possible that these minor phases, which could include oxides such as CaO and MgO, calcite (CaCO_3_), dicalcium phosphates (α-Ca_2_P_2_O_7_ and β-Ca_2_P_2_O_7_), or even α-TCP, contribute to the lack of intensity of these lines (Gomes et al., 2010).

The molar ratio of calcium to phosphorus in the M18H5 powder was estimated to be 1.53 using the following Eq. 1 (Raynaud et al., 2001):

$$\frac{Ca}{P}=\frac{\frac{10\left(100-wt\%\beta -TCP\right)}{M_{HAp}}+\frac{3wt\%\beta -TCP}{M_{WH}}}{\frac{6\left(100-wt\%\beta -TCP\right)}{M_{HAp}}+\frac{2wt\%\beta -TCP}{M_{\beta -TCP}}}$$ (1)

Where M_HAP_ and M_β-TCP_ are the molar weights of HAP and β-TCP (1004.64 and 310.18 g.mol^−1^, respectively).

### 3.4 Granulometry of the mixture

**FIGURE 7:**
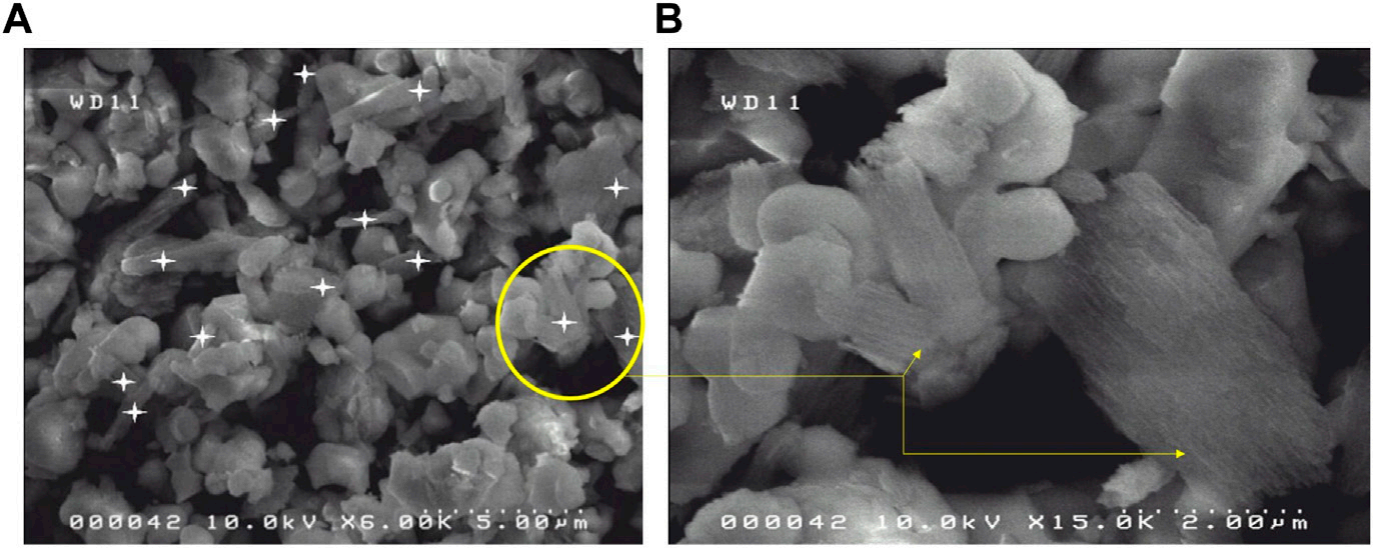
**(A)** SEM images of the mixture M18H5 and MePS. **(B)** Detail of particles of MePS and M18H5 particles.

The granulometric analysis (Table 2) shows that the average hydrodynamic diameter of the particles of M18H5 powder has the same order of magnitude as the average hydrodynamic diameter of porous silicon. Figure 7 displays a SEM image of the mixture M18H5 and MeSP. MeSP can be identified by its elongated shape and is marked with white stars. The protocol used to produce the MePS is the same as that described in Melhem et al. (2017), resulting in mesoporous silicon with columnar pores growing along the <100> directions, well-aligned, with an estimated average diameter of around 50 nm. Visible striations along the length of the marked particles correspond to columnar pores with relatively smooth walls and small branches. As the p-wafer is moderately doped (around 10^17^ cm^−3^), only one pore distribution is observed (Zhang, 2004). The porosity of the wafer is around 60%. MePS particles are generally darker in contrast than hydroxyapatite particles. Table 3 shows that the size of each type of particle is equivalent. Karbassian (2018) reported that mesoporous silicon particles can be resorbed after several days.

**TABLE 2:** Average hydrodynamic diameter of the particles of the different powders.

Powder	Average hydrodynamic diameter (nm)
M18H5	878.7
MePS	639.6

**TABLE 3:** Size of the different particles (powder M18H5 and MePS).

	Minimum size (µm)	Maximum size (µm)	Standard deviation	Average
MePS-Length	2.26	3.57	0.56	2.85
MePS-Width	0.4	2.67	0.93	1.47
M18H5	0.71	3.03	0.92	1.73

### 3.5 Assessment of powder biocompatibility

#### 3.5.1 Metabolic activity of cells exposed to calcium phosphate powder extracts

The biocompatibility of powders was assayed from powder extracts prepared as described above according guidelines of the ISO standard 10093-5. As detailed above, in order to follow these guidelines regarding mixture of M18H5 and MePS, two extracts were prepared according to the specific area of the powder mixture components. As the concentration of calcium ions in cells environment influences their behavior and due to the solubility of β-TCP and Hap at 37°C (−log (Ks) = 29.5 and 117.2, respectively (Elliott, 1994)), calcium as Ca^2+^ was dosed in the powder extracts and compared with the concentration in the complete culture medium alone. Average Ca^2+^ concentration in the complete medium was 349 ± 7.5 µM. No significant difference was found with the Ca^2+^ dosed in the powder extracts, indicating a low release of Ca^2+^ from HAp. Consequently, it can be assumed that there was a chemical equilibrium regarding Ca^2+^in these solutions.

**FIGURE 8:**
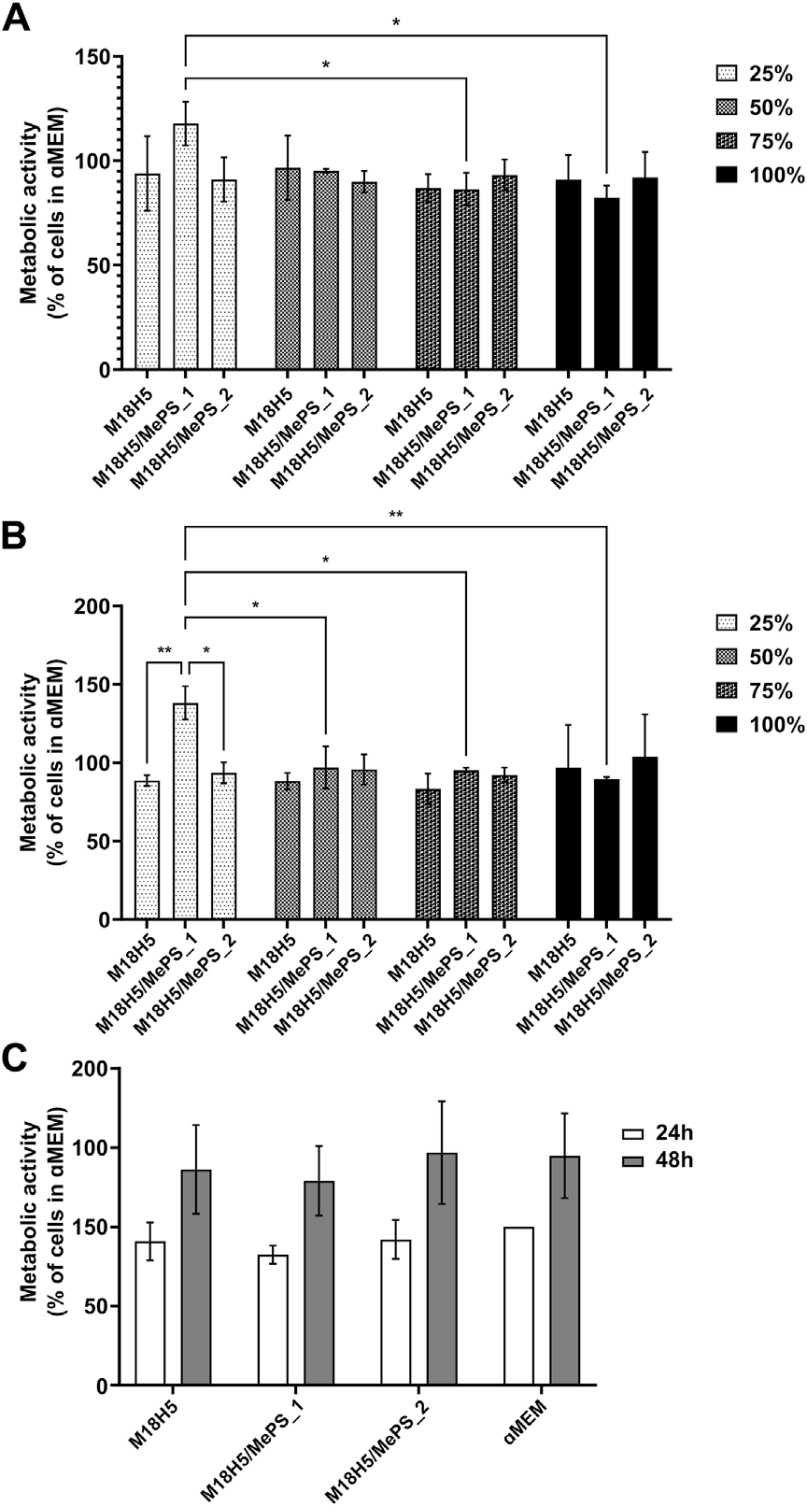
Evaluation of the metabolic activity of MC3T3-E1 cells cultured in presence of M18H5, M18H5/MePS_1 and M18H5/MePS_2 powder extracts, pure (100%) or diluted at *d* = 1/4 (75%), *d* = 1/2 (50%) and *d* = 3/4 (25%) for **(A)**: 24 h and **(B)**: 48 h. The metabolic activity is expressed as a percentage of the control, i.e. the value obtained for the cells cultured in complete culture medium (ɑMEM). **(C)**: Metabolic activity of the cell measured after 24 h and 48 h of cultivation and expressed as a percentage of the value obtained for the cell cultured in complete culture medium (ɑMEM) after 24 h as a control. Statistical analysis: ANOVA one-way followed by a Tukey *post hoc* test. *: *p* ≤ 0.05, **: *p* < 0.01. *n* = 3 independent experiments.

The MTT assay measuring the metabolic activity of the cells cultured for 24 h and 48 h in presence of the three powder extracts either pure (100%) or diluted in the complete culture medium at 1/4 (75%), 1/2 (50%) and 3/4 (25%)) is given in Figure 8. In order to evaluate the cytocompatibility (absence of cytotoxicity) of the powder extracts, the results were expressed as a percentage of activity with regards to the control, i.e. the cells cultured in the complete culture medium (Figures 8A, B). For M18H5 powders, whatever the concentration of the powder extract might be, no difference with the control was observed at both time points of 24 and 48 h.

Regarding the extracts obtained from M18H5/MePS powders (M18H5/MePS_1 and M18H5/MePS_2), no difference with M18H5 powder extract was detected at the exception of the diluted extract from the M18H5/MePS_1 powder. When the cells were cultured with 25% of the M18H5/MePS_1 powder extract in the complete culture medium, a significant increase of the cell metabolic activity was observed at both time points, 24 h (Figure 8A) and 48 h (Figure 8B). This variation can be explained either by a stimulation of the cell proliferation induced by dissolution products from M18H5/MePS powder or by an elevation of the cell metabolism (for example to meet a growing need of energy). On the Figure 8C, the results were normalized to the cells population at 24 h in order to evaluate the growth of the cell population overtime. Whatever the conditions, the cellular metabolic activity was multiplied by 1.5 over time. No significant difference was detected even regarding the condition M18H5/MePS_1 at 25% in the complete culture medium. This implies that the significant increase of the cell metabolic activity in comparison with the other conditions is not due to a stimulation of the cell proliferation leading to a higher number of metabolically active cells in the well but to a direct or indirect stimulation of the cell metabolism. The fact that only the lowest dilution induces a cell stimulation suggests that a chemical element present in the dissolution product from this sample induces a biological activity at low concentration, and that there is a threshold beyond which this stimulating effect is lost. In this respect, silicon would be a good candidate. The silicon content in 100% M18H5/MePS_1 was 53 µM (1.49 mg/L). Silicon was not detected for 100% M18H5/MePS_2 likely because the silicon concentration in this powder extract was below the detection threshold of ICP-OES. Assuming that the quantity of the released silicon is proportional to the powder amount in the culture medium, in 25% M18H5/MePS_1 the silicon content should be 1.33 µM and 0.8 µM (0.23 mg/L) for 100% M18H5/MePS_2. For this 100% M18H5/MePS_2, there was not any detected effect. Silicon substitution is widely investigated to improve the biological properties of hydroxyapatite ceramics as bone substitutes (Szurkowska and Kolmas, 2017). As a dopant in biomaterial composition, silicon was assessed to be able to induce osteogenesis and angiogenesis (Bose et al., 2013). While different responses to silicon were observed according to the cell type (Zou et al., 2008), silicon affects the *in vitro* cell behavior in a concentration range around 10 times higher than the silicon concentration measured in the 100% M18H5/MePS_1 samples (i.e. Wiens et al., 2010; Zhou et al., 2016). For example, Zhou et al., have tested concentrations in silicate of 10 and 50 mg/L on both mesenchymal stem cells and MC3T3-E1 cell line with a detectable effect on metabolic activity only after 7 days. Thus, the silicon release in the case of M18H5/MePS_2 extracts was likely too low to induce a cell response and the effect seen for diluted at 25% M18H5/MePS_1 is due to another phenomenon.

The main goal of the biological assays here was to check the biocompatibility of the extracts. According to the ISO standard 10093-5, the cytotoxicity of a compound is assessed when the metabolic activity falls down under 70% of those of the control. The metabolic activity is not altered in comparison to the control, the powder extracts are considered as not cytotoxic. Thus, while keeping in mind that a deeper insight about the effect of the more diluted M18H5/MePS_1 powder extract should be done, in the following experiments only the pure powder extracts were used.

#### 3.5.2 Cell density and proliferation

**FIGURE 9:**
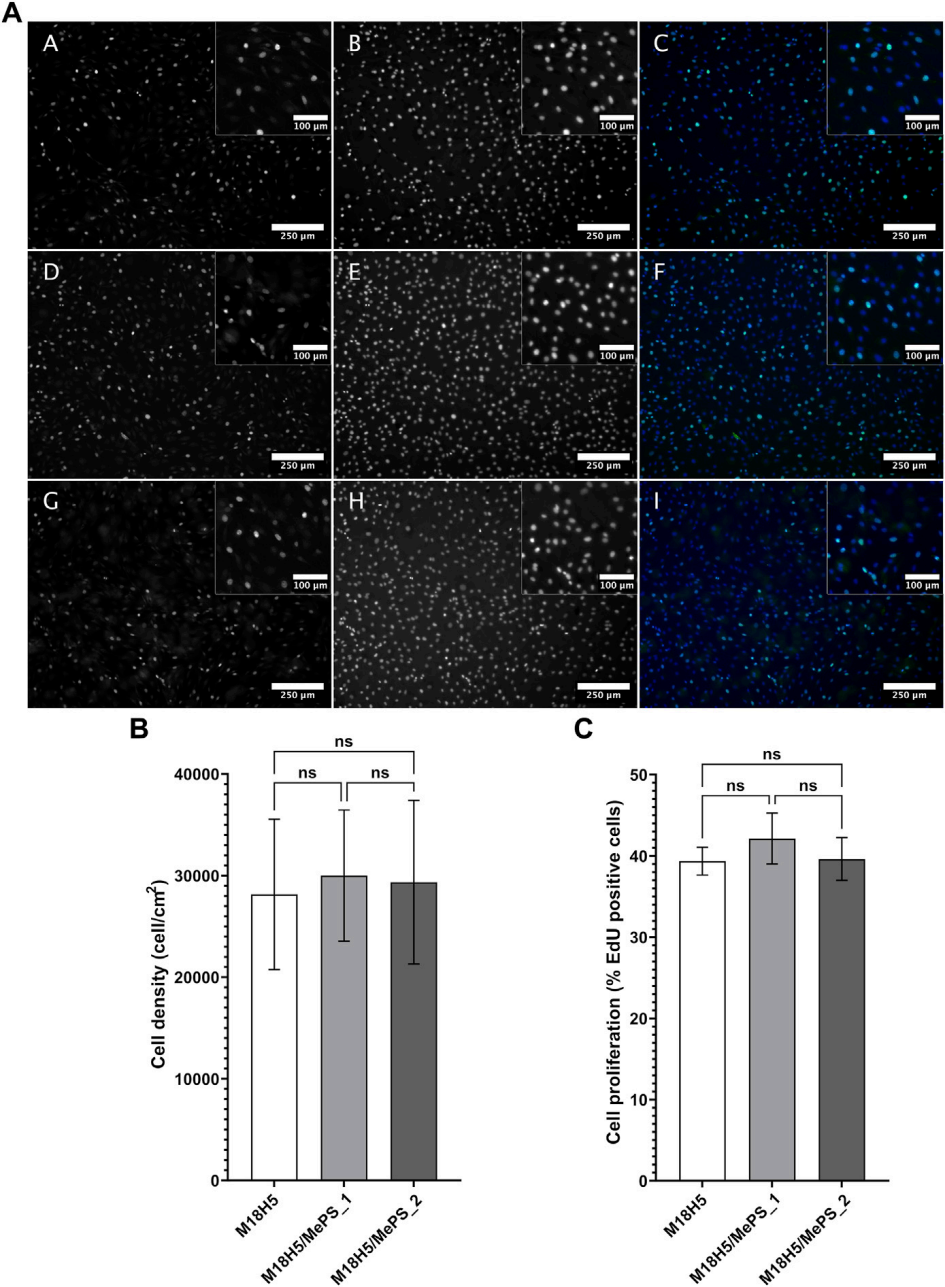
Density and proliferation rate of MC3T3-E1 cells cultured in presence of M18H5, M18H5/MePS_1 and M18H5/MePS_2 powder extracts. **(A)** Micrographs of cells cultured in presence of M18H5 (A–C), M18H5/MePS _1 (D–F) and M18H5/MePS_2 (G–I) powder extracts stained after EdU incorporation assay. A, D and (G) EdU after conjugation with AlexaFluor488; B, E and (F) nuclei stained by Hoechst 33,342; C, F and (I) merge. Scale bar: 250 µm (insets: 100 µm). Upper right: insets showing the magnification of areas in the main image **(B)** Cell density, determined by image analysis and expressed as cells/cm^2^. Statistical analysis: ANOVA one way followed by a Tukey *post hoc* test; ns: *p* > 0.05. **(C)** Cell proliferation, expressed in % of cells positive for EdU in total cell population (Hoechst 3,342 positive nuclei) determined by image analysis. Statistical analysis: Kruskall-Wallis followed by a Dunn’s *post hoc* test; ns: *p* > 0.05. *n* = 3 independent experiments.

The MTT assay showed that the MC3T3-E1 cell viability is not affected by the powder extracts and allowed to hypothesize that the proliferation rate is not affected. But the MTT assay do not test directly the proliferation rate so that it was evaluated by an EdU incorporation assay, *in situ* (Figure 9). The cells incorporated EdU during the duplication of their DNA content at the beginning of the mitosis process. Consequently, the percentage of cells having incorporated EdU (Figure 9C), obtained by image analysis after having imaged the cells in fluorescence microscopy (Figure 9A), directly reflected the proliferation rate of the cell population. Nuclei, representing the total cell number, were stained by the Hoechst 33,432. The cell populations (Figure 9A) confirmed the MTT results. The nuclei presented a healthy morphology, circular with a regular contour. The cell layers were homogeneous. Very few condensed nuclei, hallmarks of cell death, were found. The calculation of the cell density (Figure 9B) did not show any significant difference between the conditions, which confirmed the visual observations. The average density is about 27,400 cells/cm^2^ is above the cell seeding density (25,000 cells/cm^2^). After 4 h of EdU incorporation, about 40% of cells were positive (Figure 9C), and so entered the S phase of the cell cycle. The cells cultured in complete culture medium exhibited a percentage of EdU incorporation of 38.5% ± 2.0%, which cannot be considered as different from the conditions with powder extracts. The powder extracts did not alter the proliferation rate of the MC3T3-E1 cells.

#### 3.5.3 Cell morphology

**FIGURE 10:**
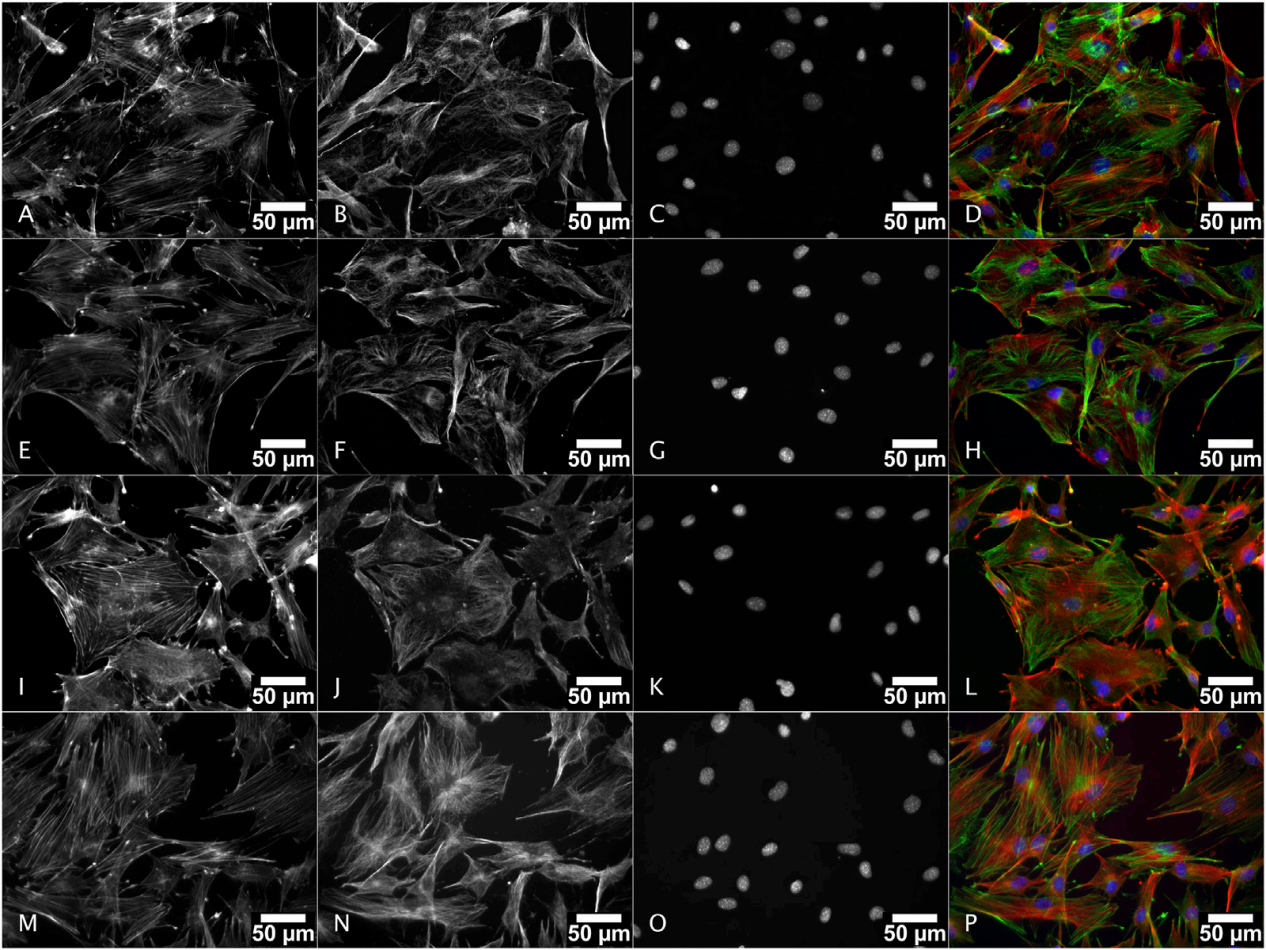
Morphology of MC3T3-E1 cells cultured in presence of M18H5, M18H5/MePS_1 and M18H5/MePS_2 powder extracts after staining of actin cytoskeleton and ɑ-tubulin. M18H5 **(A–D)**, M18H5/MePN_1 **(E–H)**, M18H5/MePN_2 **(I–L)** and complete culture medium **(M–P)**. A, E, I and M: actin cytoskeleton; B, F, J and N: ɑ-tubulin; C, G, K, and O: nuclei; D, H, L and P: merge. Scale bar: 50 μm. *n* = 3 independent experiments.

The MC3T3-E1 cell morphology in presence of powder extracts in comparison with the complete culture growth medium alone was evaluated *in situ* by fluorescence microscopy after cytoskeleton staining (Figure 10). In complete culture medium (lower row, M, N, O, P in Figure 10) the cells were well spread. Actin staining (M and green staining on P images in Figure 10) is arranged as stress fibers. At the top end of these stress fibers, the actin staining is more intense in punctual areas that might correspond to the anchoring points of the cell on the glass coverslip surface. Microtubules (N and red staining on P images in Figure 10), were distributed from the microtubule organizing center (where all microtubules converge) which is close to the nucleus, towards the cell periphery. When considering the conditions with the powder extracts, whatever the nature of the powder, the cell morphology and cytoskeleton distributions are not visually different. The cell morphology was not altered suggesting that the cell physiological behavior in terms of adhesion, orientation and related cell-cell adhesion as well as migratory aspects should not be dramatically different between these conditions.

## 4 Conclusion and outlook

The present study demonstrated that biphasic calcium phosphates can be synthesized by the solid-state chemical reaction method from a natural calcified material from the recycling of oyster shells, providing an easy and economical approach for mass producing BCP powders with MePS.

Ball milling for 18 h effectively produces calcite powders with a suitable particle size, and heat treatment at 1,050°C can transform oyster shell powders into BCP powders with a high content of β-TCP. Additionally, anodization of silicon wafers can be utilized to synthesize mesoporous silicon with pore lengths ranging from 5 to 50 nm. Furthermore, calcium phosphate mixtures with mesoporous silicon particles are biocompatible.

It would be interesting to further investigate the effects of diluted extracts prepared from powders calcium phosphates and mesoporous silicon on osteoblastic cells based on their specific surface area. Although some positive effects of silicon on bone cells have been described in the literature, it is challenging to compare these findings due to differences in methodology, silicon source, and chemical state. As such, it would be valuable to study the impact of low doses of silicon on osteoblastic cells using these specific materials.

## Data Availability

The original contributions presented in the study are included in the article/supplementary materials, further inquiries can be directed to the corresponding author.
